# *Linc-SCRG1* accelerates progression of hepatocellular carcinoma as a ceRNA of miR26a to derepress SKP2

**DOI:** 10.1186/s13046-020-01825-2

**Published:** 2021-01-09

**Authors:** Jun-Jie Hu, Cui Zhou, Xin Luo, Sheng-Zheng Luo, Zheng-Hong Li, Zi-Xin Xu, Ming-Yi Xu

**Affiliations:** Department of Gastroenterology, Shanghai General Hospital, Shanghai Jiao Tong University School of Medicine, No 100, Haining Rd, Shanghai, 200080 China

**Keywords:** Long noncoding RNA-SCRG1 (*linc-SCRG1*), microRNA-26a (miR26a), S phase kinase related protein 2 (SKP2), Hepatocellular carcinoma (HCC), Epithelial-to-mesenchymal transition (EMT)

## Abstract

**Background:**

Increasing evidence has demonstrated that long noncoding RNAs (lncRNAs) have regulatory functions in hepatocellular carcinoma (HCC). The link between *lincSCRG1* and HCC remains unclear.

**Methods:**

To explore the *lincSCRG1* regulation axis, bioinformatics, RIP and luciferase reporter assay were performed. The expressions of *lincSCRG1-miR26a-SKP2* were detected in HCC tissues and cell lines through qPCR and western blot. The functions of HCC cells were investigated through in vitro assays (MTT, colony formation, transwell and flow cytometry) and the inner effect of *lincSCRG1*-miR26a in vivo was evaluated by xenografts and liver metatstatic nude mice models.

**Results:**

*LincSCRG1* was found to be strongly elevated in human HCC tissues and cell lines. MiR26a and S phase kinase-related protein 2 (SKP2) were predicted as the target miRNA for *lincSCRG1* and the target gene for miR26a with direct binding sites, respectively. *LincSCRG1* was verified as a competing endogenous RNA (ceRNA) via negative regulation of miR26a and derepression of SKP2 in HCC cells. Both overexpression of *lincSCRG1* (ov-*lincSCRG1*) and inhibition of miR26a (in-miR26a) obviously stimulated cellular viability, colony formation, migration and proliferation of S phase cells and also significantly increased the protein levels of cyclinD1, CDK4, MMP2/3/9, Vimentin, and N-cadherin or inhibited the protein level of E-cadherin of HCC cells, while knockdown of *lincSCRG1* (sh-*lincSCRG1*) and upregulation of miR26a (mi-miR26a) had the opposite effects on HCC cells. Cotransfection of in-miR26a or overexpression of SKP2 (ov-SKP2) with sh-*lincSCRG1* could rescue the anticancer functions of sh-*lincSCRG1*, including suppressing proliferation and migration of HCC cells. Additionally, sh-*lincSCRG1* could effectively inhibit the growth of subcutaneous xenograft tumours and lung metastasis, while the anticancer effect of sh-*lincSCRG1* could be reversed by cotransfection of in-miR26a.

**Conclusions:**

*LincSCRG1* acts as a ceRNA of miR26a to restrict its ability to derepress SKP2, thereby inducing the proliferation and migration of HCC cells in vitro and in vivo. Depletion of *lincSCRG1* could be used as a potential therapeutic approach in HCC.

**Supplementary Information:**

The online version contains supplementary material available at 10.1186/s13046-020-01825-2.

## Background

Hepatocellular carcinoma (HCC) is regarded as the most common primary malignancy of the liver and a major cause of mortality [1]. In addition, HCC is not only resistant to conventional therapies but also has a high recurrence rate [2]. Thus, in view of its malignancy and the fact that its pathogenesis is still a mystery, it is necessary to elucidate the essential pathogenesis of HCC for the identification of novel therapeutic targets.

Long noncoding RNAs (lncRNAs) are a class of functional noncoding RNA molecules with a transcript greater than 200 nucleotides in length [3]. However, the exact biotic mechanisms and functions of most lncRNAs in HCC remain largely unknown. To date, nearly 30 deregulated lncRNAs have been identifiedas being associated with HCC [4]. Thus, lncRNAs affect cell proliferation, apoptosis and metastasis and regulatethe tumour microenvironment in HCC, eventually causing tumour development [5].

We previously showed that *lincSCRG1* was significantly upregulated in human cirrhotic livers and involved in accelerating liver fibrosis [6]. Based on this, we illustrate the role of *lincSCRG1* in HCC. Our findings revealed that the *lincSCRG1*/miR26a/SKP2 axis is a novel regulatory mechanism implicated in HCC progression, which provides a potential treatment strategy for HCC.

## Materials and methods

### Clinical specimens

Six patients with HCC were enrolled (4 males, 2 females, age range 30–75 years old) in the study. Six paired HCC and the corresponding adjacent liver tissues were obtained from the surgical specimen archives of Shanghai General Hospital. The diagnosis and clinical stage of HCC was based on clinical and histological diagnostic criteria [7]. All enrolled patients provided written informed consent and this study was approved by the ethics committee of Shanghai General Hospital.

### Animal experiment

A total of 18 BALB/c nude mice (6–8 weeks old, male, 18 ± 2 g) were used (Cavens Laboratory Animal Center, Changzhou, China). All mice were randomly divided into 2 groups with SNU-387 cells (2 × 10^6^ cells/100 μl, 100 μl/mouse) injected subcutaneously for the tumour xenograft model or through the tail vein for the metastasis model (each group, *n* = 9). Then, mice in each group were randomly divided into 3 subgroups (each subgroup, *n* = 3). Lentivirus (lentivirus titre, 10^8^ TU/ml, 20 μl/mouse) packed with a short hairpin RNA (shRNA) of *lincSCRG1*(sh-*lincSCRG1*, Invitrogen, Carlsbad, CA and HanbioBiotechnology, Shanghai, China) to silence *lincSCRG1*, a shRNA negative control (sh-NC, Invitrogen), or sh-*lincSCRG1* + miR26a inhibitor (sh-*lincSCRG1* + in-miR26a, GenePharma Co., Ltd., Shanghai, China) to silence *lincSCRG1*, and miR26a was injected every week for 3 continuous weeks. The sequences of the oligonucleotides used are listed in Suppl. Table 1. After 31 days, all nude mice were euthanized. The subcutaneous tumours were excised, photographed, and weighed, and the tissues were stained with immunohistochemistry (IHC) for Ki-67. The lung tissues of the metastasis model were stained with haematoxylin & eosin (H&E). All these procedures followed the guidelines and were approved by the Ethics Committee of Shanghai General Hospital.

### Cell line culture

The human liver cell line (LO2), mouse liver cell line (AML-12) and HCC cell lines (HepG2, Hep3B, HCCLM3, SNU-387) were used. Primary hepatocytes (PHCs) were isolated from wide-type (WT) C57BL/6 mice using a two-step collagenase digestion method. Cells were cultured in Dulbecco’s modified Eagle’s medium (DMEM) or M199 medium (Gibco, Grand Island, NY).

### Lentivirus construction and transfection

Expression vectors encoding homo sh-*lincSCRG1* were used to knockdown genes,and vectors encoding homo ov-*lincSCRG1* were used to overexpress *lincSCRG1* (Hanbio Biotechnology). Lentivirus vectors of pLVTHM and pLV-IRES-DsRed were used to construct the expression plasmids. The virus supernatant was harvested from 293 T cells. Then, SNU-387 or Hep3B cells were transfected with lentivirus for 72 h. The efficiency of infection was verified by fluorescence microscopy. The sequences of the oligonucleotides used are listed (Suppl. Table 1).

The full-length cDNA fragment (1275 bp) of SKP2 (S phase kinase related protein 2) (NM_005983, Suppl. Table 1) was PCR amplified and used for genetic transformation for the overexpression experiments.

MiR26a mimic (mi-miR26a), miRNA negative control (mi-NC), miR26a inhibitor (in-miR26a) or miRNA inhibitor negative control (in-NC) was used to overexpress or knockdown miR26a (GenePharma Co., Ltd., Suppl. Table 1). SNU-387 or Hep3B cells were transfected using Lipofectamine 3000 (Invitrogen) according to the manufacturer’s instructions. Then, the transfected cells were cultured (37 °C, 5% CO2) for 24 h.

### Quantitative real-time PCR (qPCR)

Total RNA was extracted from tissue and cells using TRIzol reagent (Invitrogen) according to the instructions. QPCR was performed using a SYBR Green PCR Kit (Applied Biosystems, Carlsbad, CA) and an ABI 7900HT Fast Real-Time PCR System (Applied Biosystems). The sequences of the primers used are listed in Suppl. Table 2.

### Western blot analysis

Western blotting was performed using the following antibodies: anti-cyclin D1 (1:2000), anti-CDK4/6 (cyclin-dependent kinases 4/6,1:2000), anti-MMP2/3/9 (matrix metalloproteinases 2/3/9, 1:1000–2000), anti-E-cadherin (1:500), anti-N-cadherin (1:100), anti-vimentin (1:2000), anti-SKP2 (1:500), and anti-GAPDH (1:2500), and the secondary antibody HRP-IgG (1:10,000) was used (Abcam, Cambridge, MA). The details of the antibodies are displayed in Suppl. Table 3.

### MTT assay

The 3-(4,5-dimethylthiazol-2-yl)-2,5-diphenyltetrazolium bromide (MTT) assay was employed to examine the proliferation of cells. The optical density (OD) value was measured by a spectrophotometric plate reader (Thermo, Waltham, MA) at 570 nm.

### Colony formation assay

The cells were digested with 0.25% trypsin to generate a single cell suspension for counting. Cell suspensions of each group were inoculated on a dish (200 cells/dish) to continue culture (5% CO2, 37 °C) until the colonies were visible. The cells were washed with PBS solution (pH 7.4) twice and fixed with methanol for 15 min, and the solution was discarded. Then the samples were stained with crystal violet for 10 min, washed with running water, and dried in air at room temperature. The number of colonies with ≥50 cells was counted under the microscope.

### Flow cytometry

For analysis of the cell cycle, cells were seeded in 6-well plates at 5 × 10^4^ cells per well. After transfection for 48 h, the cells were trypsinized and fixed in 70% ethanol at − 20 °C for 24 h. Then, the cells were stained using BD Pharmingen™ PI/RNase staining (BD Pharmingen, San Diego, CA). The cell cycle distribution was analysed using an Accuri C6 flow cytometer (Becton Dickinson and Company, Franklin Lakes, NJ). The data were analysed using ModFit LT software.

### Transwell assay

The cells (1 × 10^5^ cells/well) were seeded in an incubator (37 °C, 5% CO2) to cultivate for 24 h in a Transwell chamber (Corning Incorporation, Corning, NY) containing serum-free medium in the upper chamber and DMEM containing 10% foetal bovine serum in the lower chamber. Cells migrating to the lower chamber were fixed with 4% paraformaldehyde, stained with 0.5% crystal violet for 15 min, washed with PBS 3 times, and photographed under a microscope.

### RNA immunoprecipitation (RIP)

RIP experiments were performed according to the manufacturer’s instructions (Magna RIP RNA-Binding Protein IP Kit, Millipore, Billerica, MA). Briefly, cells were lysed in 100 μl of RIP lysis wash buffer. Whole cell extract (50 μl) was added to mouse IgG (Abcam) and anti-Ago2 (Santa Cruz Biotechnology, Inc., Santa Cruz, CA) in RIP buffer containing protein A/G magnetic bead-bound complexes. The RNA levels in the precipitates were determined by qPCR.

### Luciferase assay

The wild-type or mutant fragments of homo *lincSCRG1* or SKP2 3′UTR containing putative binding sites for miR26a were synthesized and cloned into the pGL3-Basic reporter vector (Promega, Madison, WI). SNU-387 or Hep3B cells were harvested after transfection for 24 h, and luciferase activity was detected by a Dual Luciferase Reporter Assay Kit (Beyotime, Shanghai, China) according to the manufacturer’s instructions.

### Statisticalanalysis

All experiments were repeated three times. The bar and curve graphs show the mean and standard deviation. Data were analysed using ANOVA. All statistical analyses were performed using SPSS 25.0 (SPSS, Version X; IBM, Armonk, NY). A *p* value < 0.05 was considered statistically significant.

## Results

### The expression of *lincSCRG1* was substantially upregulated in both human liver tissues and HCC cell lines

**Fig. 1:**
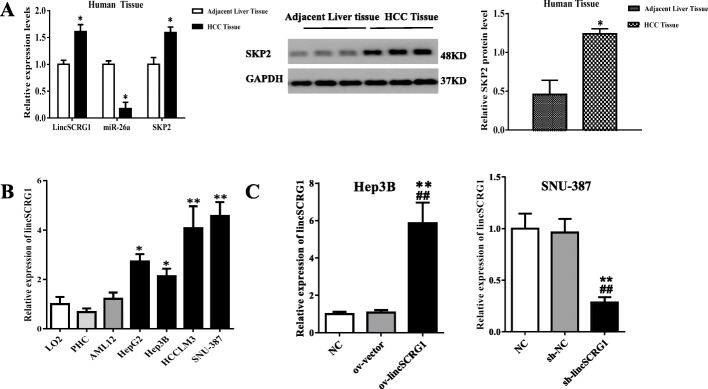
Expression of *lincSCRG1* was strongly upregulated in both human tissues and cell lines of HCC. **a** Relative *lincSCRG1*, miR26a and SKP2 expression was detected by qPCR (each group *n* = 6) and the levels of SKP2 proteins were detected by western blot (each group *n* = 3) in human HCC tissues and adjacent liver tissues. **b** The expression of *lincSCRG1*was examined in different cell lines (hepatocyte cell line [LO2, AML12], primary hepatocyte [PHC] and HCC cell lines [HepG2, Hep3B, HCCLM3, SNU-387]) by qPCR. **c** The overexpression efficiency of ov-*lincSCRG1* in Hep3B cells and the interference efficiency of sh-*lincSCRG1* in SNU-387 cells were identified by qPCR. In (**a**), ^*^indicates vs. the adjacent liver tissue group, *p <* 0.05; In (**b**)^*/**^indicates vs. the LO2 group (^*^, *p <* 0.05, ^**^, *p <* 0.01); In (**c-d**) ^**^indicatesvs. The NC group, *p <* 0.01, ^##^indicatesvs. The ov-vector/sh-NC group, *p <* 0.01

To explore the role of HCC-related lncRNAs, we focused on *lincSCRG1* (XLOC_004166; lnc-Hand2–2:1; located on chr4:173453013–173,520,960; transcript length 3118 bp). In our previous study, we found that *lincSCRG1* distinctly increased along with the progression of liver fibrosis in both human liver tissues and hepatic stellate cells (HSCs) [6]. Based on the premise that most liver cancer develops from liver cirrhosis, we hypothesized that *lincSCRG1* also plays a key role in HCC progression. PCR analysis indicated that *lincSCRG1*expression was clearly elevated in the human HCC tissues compared to the adjacent liver tissues (each group *n* = 6, *p* < 0.05, Fig. 1a). *LincSCRG1* was significantly upregulated in HCC cell lines, including HepG2, Hep3B, HCCLLM3, and SNU-387, compared to the LO2 or AML12 cells or PHCs (*p* < 0.05, Fig. 1b). In the HCC cell lines, the expression of *lincSCRG1*was highest in the SNU-387 cell line and lowest in the Hep3B cell line (Fig. 1b). Therefore, we concluded that *lincSCRG1* is increased in human HCC liver tissues and cells.

### Overexpression of *lincSCRG1* could dramatically promote the cell proliferation and migration of HCC in vitro

Since SNU-387 cells exhibited the highest expression of *lincSCRG1,* while Hep3B cells displayed the lowest expression among HCC cell lines, we selected them for the following in vitro study. To evaluate the molecular function of *lincSCRG1*, we established sh*-lincSCRG1* (in SNU-387 cells) and ov-*lincSCRG1* (in Hep3B cells) cell lines. The overexpression efficiency of ov-*lincSCRG1* in Hep3B cells (ov-*lincSCRG1*: 5.86-fold vs. NC; 5.44-fold vs. ov-vector) and interference efficiency of sh-*lincSCRG1* in SNU-387 cells (sh-*lincSCRG1*: 71.6% vs. NC; 70.5% vs. sh-NC) were identified (Fig. 1c).

**Fig. 2:**
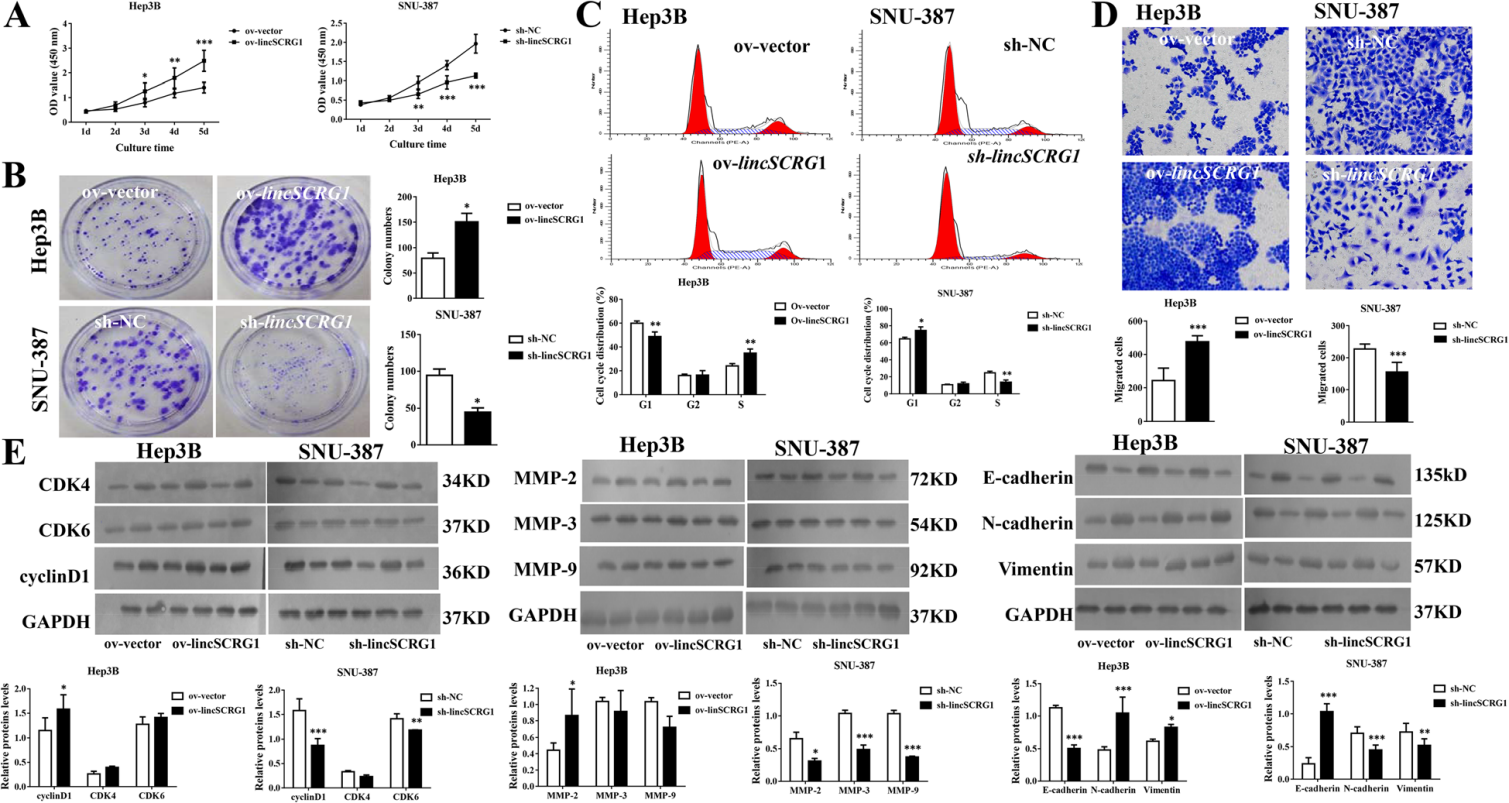
Overexpression of *lincSCRG1 *dramatically promoted HCC cell proliferation and migration in vitro. Sh*-lincSCRG1*, sh-NC (in SNU-387 cells), ov-*lincSCRG1* and ov-vector (in Hep3B cells) cell lines were established. **a** Cell viabilitywas examined by MTT assays. **b** Oncogenic survival wasassessed by colony formation assays. **c** Cell cycle proliferation was evaluated by flow cytometry. **d** Migration was determined by transwell assays. **e** Cell cycle-related proteins (CKD4/6 and cyclinD1) and EMT-related proteins (MMP-2/3/9, E-cadherin, N-cadherin and Vimentin) were examined by western blot analysis. In (**a-e**), ^*/**/***^indicatesvs. The ov-vector/sh-NC group (^*^, *p <* 0.05, ^**^, *p <* 0.01, ^***^, *p <* 0.001)

Ov-*lincSCRG1* significantly increased cell viability from Day 3 to 5 in the MTT assay and promoted cell proliferation in the colony formation assay of Hep3B cells, while sh-*lincSCRG1* strongly inhibited the cellular viability and proliferation of SNU-387 cells (Fig. 2a/b). A lower frequency of cells in G1 phase and a higher frequency of cells in S phase were observed in the ov-*lincSCRG1* Hep3B cells than in the ov-NC Hep3B cells, while the opposite results were observed in the sh-*lincSCRG1* SNU-387 cells (*p* < 0.05, Fig. 2c). However, neither ov-*lincSCRG1* nor sh-*lincSCRG1* influenced the percentage of cells in G2 phase (Fig. 2c). Consistently, ov-*lincSCRG1* obviously increased the pro-proliferative protein cyclin D1 in Hep3B cells, while sh*-lincSCRG1* inhibited the protein levels of cyclin D1 and CKD6 in SNU-387 cells (Fig. 2e). Furthermore, ov-*lincSCRG1* significantly increased the migration of Hep3B cells, while sh-*lincSCRG1* had the opposite effect in SNU-387 cells (Fig. 2d). The protein levels of MMP-2/3/9 (epithelial to mesenchymal transition [EMT]-related markers) were significantly decreased in the sh*-lincSCRG1* SNU-387 cells, but only MMP-2 was markedly increased in the ov-*lincSCRG1* Hep3B cells (Fig. 2e). For the adhesin proteins, ov-*lincSCRG1* decreased the levels of E-cadherin but elevated the levels of N-cadherin and Vimentin in Hep3B cells, while sh-*lincSCRG1* produced the opposite effects in SNU-387 cells (Fig. 2e). These results indicated that upregulation of *lincSCRG1* could promote the proliferation, migration and EMT of HCC cells, while silencing of *lincSCRG1* could have the opposite effects.

### *LincSCRG1* negatively regulates miR26a by acting as a ceRNA

**Fig. 3:**
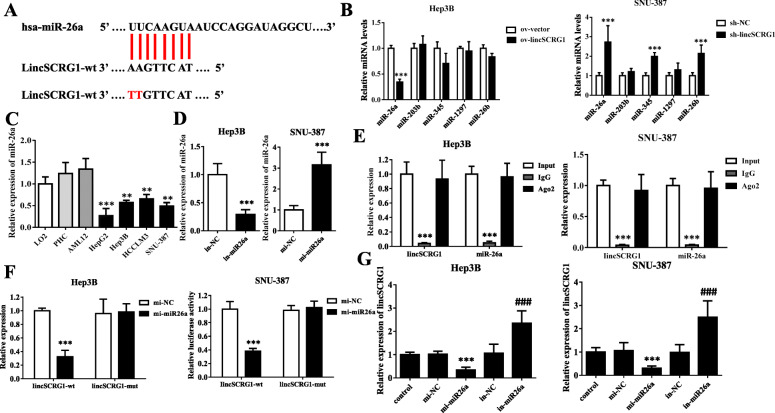
*LincSCRG1* negatively regulates miR26a by acting as a ceRNA. **a** The binding sites of miR26a on *lincSCRG1*are represented. **b** qPCR was performed to evaluate the expression of miR26a/b, 203b, 345 and 1297 in *ov-lincSCRG1* Hep3B cells and sh-*lincSCRG1* SNU-387 cells. **c** The expression of miR26a was examined in different cell lines (LO2, AML12, PHC, HepG2, Hep3B, HCCLM3, SNU-387) by qPCR. **d** Overexpression efficiencies of mi-miR26a and mi-NC were examined in the SNU-387 cell line, and interference efficiencies of in-miR26a and in-NC were examined in the Hep3B cell line by qPCR. RIP experiments (**e**) and luciferase reporter assays (**f**) were performed to demonstrate that miR26a was bound to *lincSCRG1* in Hep3B and SNU-387 cells. **g** qPCR analysis showed that miR26a suppressed the expression of *lincSCRG1*, which could be promoted by in-miR26a. In **b**, ^***^indicatesvs. The ov-vector/sh-NC group, *p <* 0.001; In (**c**), ^*/**/***^indicatesvs. The LO2 group (^*^, *p <* 0.05, ^**^, *p <* 0.01, ^***^, *p <* 0.001); In (**d, f**), ^***^indicatesvs. The mi-NC/in-NC group, *p <* 0.001; In (**e**), ^***^indicatesvs. The IgG group, *p <* 0.001; In (**g**) ^***^indicatesmi-miR26a vs. the mi-NC group, *p <* 0.001; ^####^indicatesin-miR26a vs. the in-NC group, *p <* 0.001

LncRNAs are known to act as molecular sponges of miRNAs, also called competing endogenous RNAs (ceRNAs) for specific miRNAs, to exert their function [8, 9]. Therefore, we hypothesized that *lincSCRG1* might also interact with miRNAs as a ceRNA in HCC. The interaction probabilities between *lincSCRG1* and miRNAs were assessed through the online software miRDB (http://mirdb.org/miRDB/index.html) , which showed that 50 miRNAs might be regulated by *lincSCRG1* (Suppl. Table 4). Then, 7 miRNAs (miR-1297, 4465, 26a/b, 4779, 4778, 345, 203b) were identified due to low expression in tumour tissues. By searching the literature, we found that 5 miRNAs (miR26a/b, 345, 203, 1297) were reported to be involved in HCC and selected them for further verification by qPCR. Only the expression of miR26a was clearly increased in the *sh-lincSCRG1* SNU-387 cells compared to the sh-NC cells (2.7-fold, *p* < 0.05) but significantly decreased in the ov-*lincSCRG1* Hep3B cells compared to the ov-NC cells (0.34-fold, *p* < 0.05, Fig. 3b). The data revealed that *lincSCRG1* negatively regulated miR26a in HCC cells. In addition, Lin showed that lncRNA DLGAP1-AS1 could act as a ceRNA of miR26a to promote HCC development [10]. Taken together, these results suggest that miR26a is a target ceRNA of *lincSCRG1* for further elucidation. Through bioinformatic analysis of miRDB, predicted binding sites between miR26a and *lincSCRG1*were identified (Fig. 3a). The expressions of miR26a were significantly down-regulated in the human HCC tissues compared to the adjacent liver tissues (each group *n* = 6, *p* < 0.05, Fig. 1a). In addition, substantially decreased expression of miR26a was observed in these 4 HCC cell lines compared to LO2 or AML12 cells or PHCs, so we selected the Hep3B and SNU-387 cell lines for further study (Fig. 3c). To downregulate or upregulate miR26a, we transfected miR26a mimics, inhibitors and their individual negative controls (mi-miR26a and mi-NC, in-miR26a and in-NC) into SNU-387 and Hep3B cells (Fig. 3d). Furthermore, RIP assays showed that both *lincSCRG1* and miR26a were enriched in the precipitated complex loaded with Ago2 antibodies but not IgG antibodies (Fig. 3e). The luciferase activity in the SNU-387 and Hep3B cells cotransfected with mi-miR26a and *lincSCRG1*-wt (wild type of *lincSCRG1)* was significantly reduced compared withthat in the cells transfected with mi-NC and *lincSCRG1*-wt, while the luciferase activity in the mi-miR26a and *lincSCRG1*-mut (mutant type of *lincSCRG1*) transfection group and mi-NC and *lincSCRG1*-mut transfection group had no obvious difference (Fig. 3f). To further explore the competitive relationship of miR26a and *lincSCRG1*, we detected the relative expression of *lincSCRG1* when SNU-387 and Hep3B cells were treated with miR26a mimics or inhibitors. Mi-miR26a strongly inhibited *lincSCRG1* expression by approximately 70%, while in-miR26a counteracted this effect by approximately 2-fold compared to individual negative controls in both cell lines (Fig. 3g). Therefore, our data revealed that *lincSCRG1* has binding sites with miR26a and negatively regulates miR26a as a ceRNA.

### MiR26a is negatively correlated with the proliferation and migration of HCC cells in vitro

**Fig. 4:**
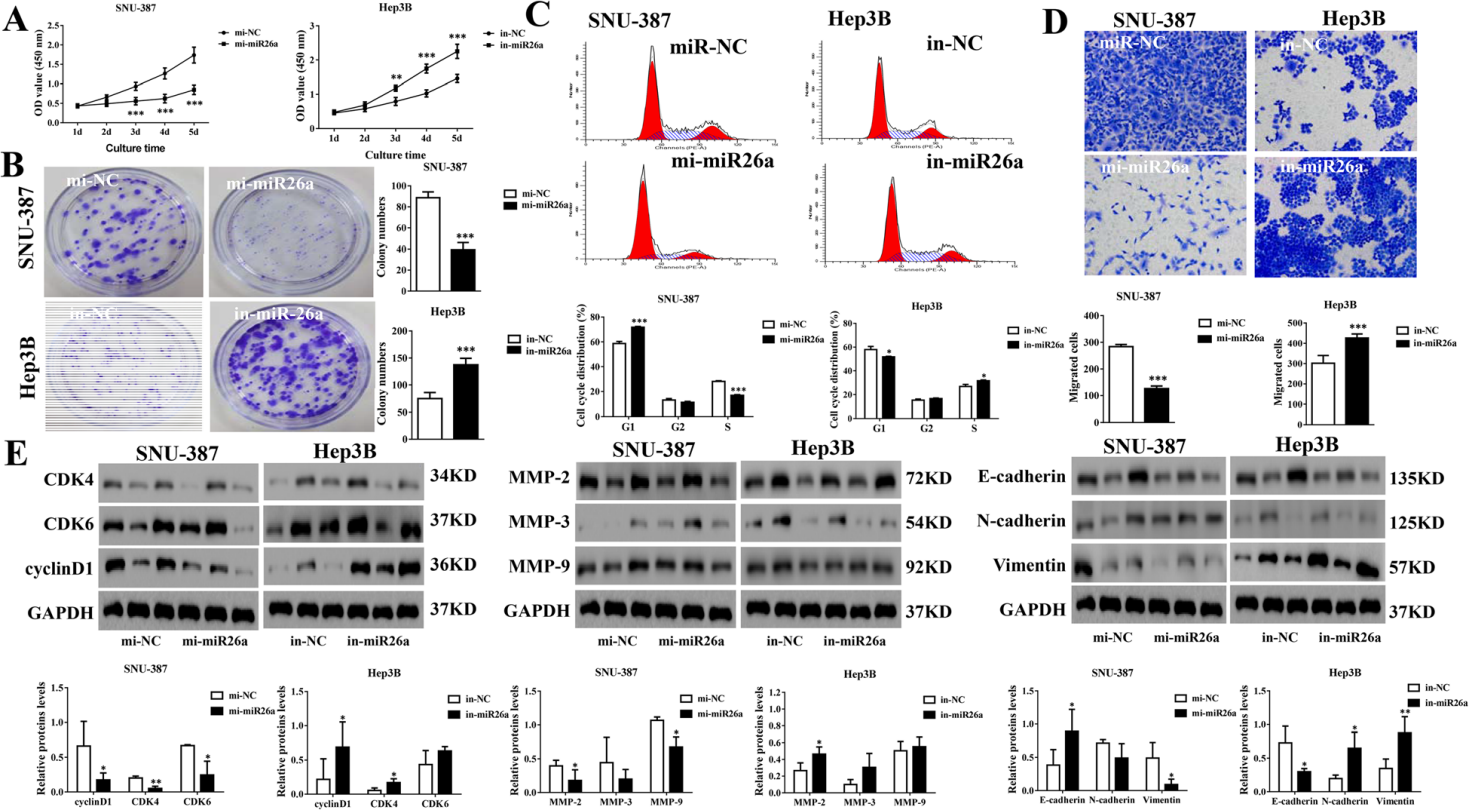
MiR26a is negatively correlated with the proliferation and migration of HCC in vitro. Mi-miR26a, mi-NC (in SNU-387 cells), in-miR26a and in-NC (in Hep3B cells) cell lines were established. **a** Cell viabilitywas examined by MTT assays. **b** Oncogenic survival wasassessed by colony formation assays. **c** Cell cycle proliferation was evaluated by flow cytometry. **d** Migration was determined by transwell assays. **e** Cell cycle-related proteins (CKD4/6 and cyclinD1) and EMT-related proteins (MMP-2/3/9, E-cadherin, N-cadherin and Vimentin) were examined by western blot analysis. In (**a-e**), ^*/**/***^indicatesvs. The mi-NC/in-NC group (^*^, *p <* 0.05, ^**^, *p <* 0.01, ^***^, *p <* 0.001)

To explore the role of miR26a in HCC in vitro, we established 2 groups of SNU-387 cells (mi-miR26a and mi-NC group) and Hep3B cells (in-miR26a and in-NC group). Upregulation of miR26a obviously inhibited cell viability from Day 3 to Day 5 in the MTT assay, suppressed cell proliferation in the colony formation assay, increased the frequency of cells in G1 phase and decreased the frequency of cells in S phase in flow cytometry analysis, and decreased the migrating number of cells in the transwell assay of SNU-387 cells, while downregulation of miR26a resulted in opposite effects on Hep3B cells (Fig. 4a-d). The levels of pro-proliferative proteins (cyclin D1, CDK4) and EMT-related proteins (MMP2, Vimentin) were clearly decreased, while EMT-related proteins (E-cadherin) were evidently elevated in the mi-miR26a SNU-387 cells (Fig. 4e). Opposite effects could be observed in the in-miR26a Hep3B cells (Fig. 4e). These data illustrated that overexpression of miR26a probably suppresses cell proliferation and migration of HCC cells.

### SKP2 is the direct target gene of miR26a, and the *lincSCRG1*/miR26a/SKP2 regulatory axis was identified in HCC in vitro

**Fig. 5:**
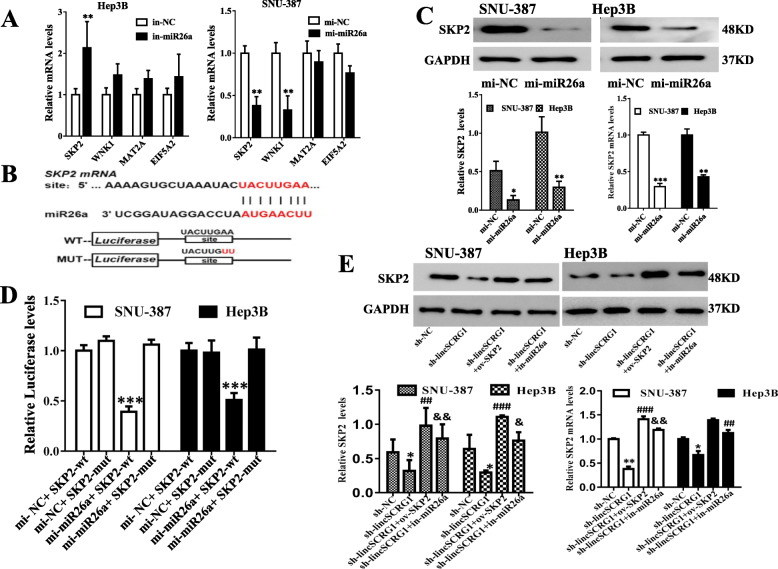
SKP2 is the direct target gene of miR26a in HCC in vitro. **a** qPCR was performed to evaluate the expression of SKP2, WNK1, MAT2A and EIF5A2 in Hep3B cells and SNU-387 cells. **b** SKP2 consensus binding site sequence and miR26a sequence alignment. The binding sequence is shown. **c** The SKP2 mRNA and protein expression was examined in SNU-387 cells and Hep3B cells treated with mi-miR26a. **d** Luciferase activity was strongly decreased when miR26a and SKP2-wt were cotransfected in SNU-387 cells and Hep3B cells in a luciferase reporter assay. **e** Sh-NC, sh-*lincSCRG1*, sh-*lincSCRG1* + ov-SKP2 and sh-*lincSCRG1* + in-miR26a groups were established in SNU-387 and Hep-3B cell lines. The expression of SKP2 mRNA and proteins wasstrongly impeded by sh-*lincSCRG1*, but this effect was reversed by in-miR26a and ov-SKP2 in SNU-387 cells and Hep3B cells. In (**a-c**) ^**/***^indicates vs. the mi-NC/in-NC group (^**^, *p <* 0.01, ^***^, *p <* 0.001); In (**d**) ^***^indicatesvs. The mi-miR26a + SKP2-mut,*p <* 0.001. In (**e**) ^**^indicates vs. the sh-NC group, *p <* 0.01; ^##/###^indicates vs. the sh-*lincSCRG1* group (^##^, *p <* 0.01, ^###^, *p <* 0.01); ^&&^indicatesvs. The sh-*lincSCRG1* group, *p <* 0.01

To probe the molecular mechanisms underlying miR26a, we used miRNA target prediction programmes (TargetScan, miRDB and miRcode) and the Disgenet database to search for the candidate targets of miR26a. A total of 8 potential target genes that were identified to participate in HCC progression were selected by bioinformatics analysis. Among them, 4 target genes, SKP2, WNK1 (with-no-lysine kinase 1), MAT2A (methionine adenosyltransferase 2A) and EIF5A2 (eukaryotic translation initiation factor 5 A2), have never been reported to be regulated by miR26a. The mRNA and protein levels of SKP2 were strongly elevated in the human HCC tissues compared to the adjacent liver tissues (each group of PCR *n* = 6, each group of western blot *n* = 3, *p* < 0.05, Fig. 1a). QPCR analysis verified that only the expression of SKP2 was clearly increased in the in-miR26a HCC cells compared to the in-NC cells, while it was significantly decreased in the mi-miR26a cells compared to the mi-NC cells (Fig. 5a). While the relationship between SKP2 and miR26a is still unknown, we identified SKP2 as a target gene for further study. SKP2 is the F-box protein of the E3 ubiquitin ligase complex and is responsible for targeted recognition and degradation of different cyclin-dependent kinase inhibitors [11]. Furthermore, recent studies have shown that SKP2 plays an oncogenic role in promoting tumour cell growth and migration in HCC [12]. According to the search results, SKP2 has eight binding sites in the 3′-untranslated region (3’UTR) with miR26a (Fig. 5b), which indicated their higher binding affinity. To confirm that SKP2 is the downstream target of miR26a, we established a miR26a overexpression (mi-miR26a) group and a negative control (mi-NC) group in both the SNU-387 and Hep3B cells. In the mi-miR26a cells, the expression of SKP2 proteins was reduced by approximately 60–70% compared with that in the mi-NC cells in both cell lines (Fig. 5c, Fig. S1A). Consistently, mi-miR26a distinctly downregulated the SKP2 mRNA levels in both cell lines (Fig. 5c, Fig. S1A). The recognition sites or the mutant sequences of SKP2 (wild type or mutant type of SKP2 [SKP2-wt/−mut]) were subcloned into luciferase reporter plasmids in both cell lines treated with miR26a mimics or negative control. Only cotransfection with SKP2-wt and overexpression of miR26a decreased luciferase activity (Fig. 5d). Finally, to validate the regulatory function of *lincSCRG1* via the miR26a/SKP2 axis, we treated SNU-387 and Hep3B cells with sh-*lincSCRG1*, sh-*lincSCRG1* + ov-SKP2, sh-*lincSCRG1* + in-miR26a and sh-NC. Sh-*lincSCRG1* substantially downregulated SKP2 at both the mRNA and protein levels, while cotransfection with ov-SKP2 or in-miR26a abolished this effect (Fig. 5e, Fig. S1B). Thus, miR26a could bind to the target sites and directly downregulate SKP2. Then, we demonstrated the regulatory mechanism of the *lincSCRG1*/miR26a/SKP2 axis in HCC cells.

### *LincSCRG1* promotes cell proliferation and migration of HCC via regulating the miR26a/SKP2 axis in vitro

Furthermore, to elucidate the effect of the *lincSCRG1*/miR26a/SKP2 axis on cell growth and migration in HCC, we treated SNU-387 and Hep3B cells with sh-*lincSCRG1*, sh-*lincSCRG1* + ov-SKP2, sh-*lincSCRG1* + in-miR26a and sh-NC.

**Fig. 6:**
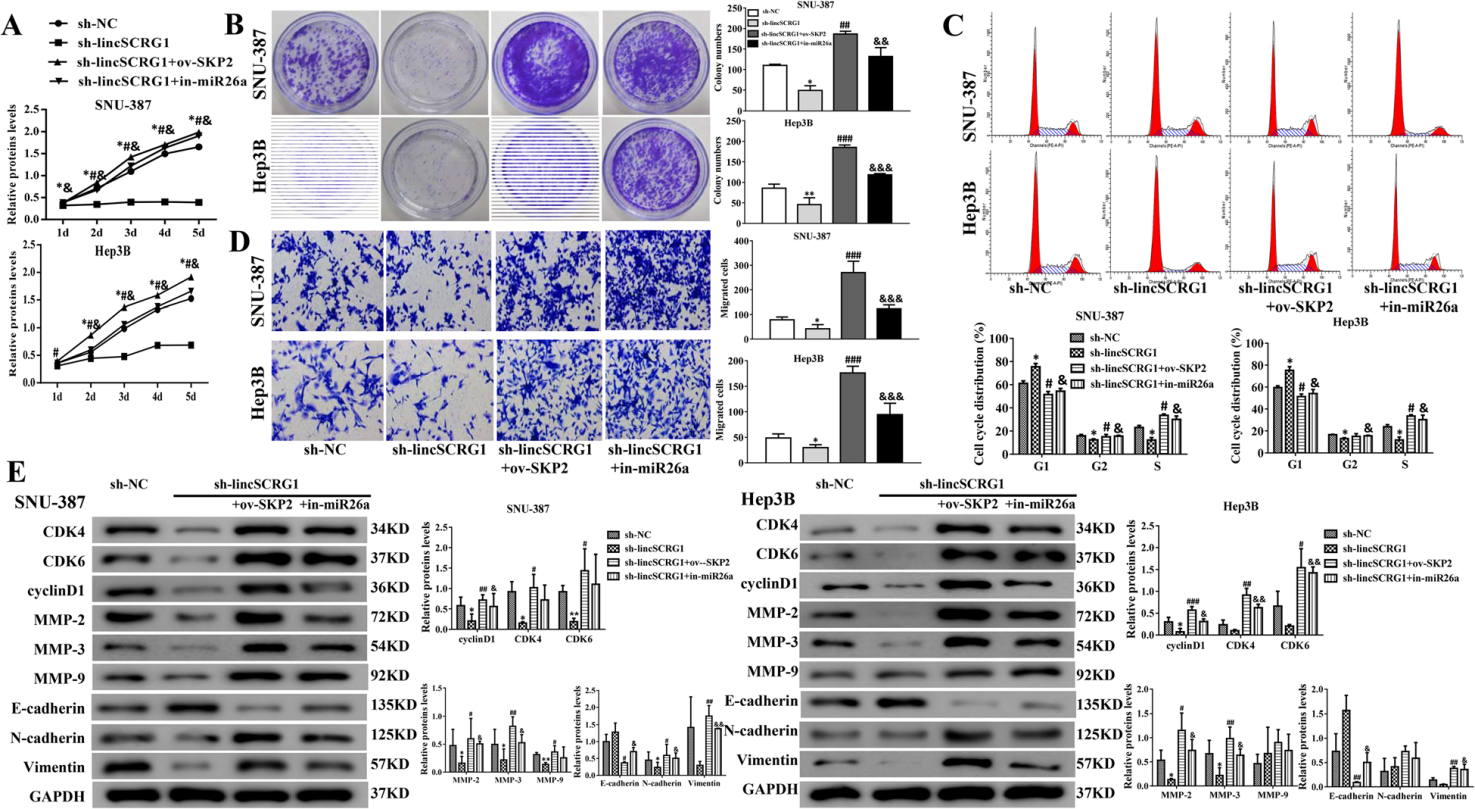
*LincSCRG1* promotes cell proliferation and migration of HCC via regulating the miR26a/SKP2 axis in vitro. Sh-NC, sh-*lincSCRG1*, sh-*lincSCRG1* + ov-SKP2 and sh-*lincSCRG1* + in-miR26a groups were established in SNU-387 and Hep-3B cell lines. **a** Cell viabilitywas examined by MTT assays. **b** Oncogenic survival wasassessed by colony formation assays. **c** Cell cycle proliferation was evaluated by flow cytometry. **d** Migration was determined by transwell assays. **e** Cell cycle-related proteins (CKD4/6 and cyclinD1) and EMT-related proteins (MMP-2/3/9, E-cadherin, N-cadherin and Vimentin) were examined by western blot analysis. In (**a - d**) ^*/#/&^indicatesthe sh-*lincSCRG1* vs. sh-NC group, the sh-*lincSCRG1* + ov-SKP2 vs. sh-*lincSCRG1* group, and the sh-*lincSCRG1* + in-miR26a vs. sh-*lincSCRG1* group, respectively (*n* = 6). ^* /#/&^, *p <* 0.05, ^**/##/&&^, *p <* 0.01, ^***/###/&&&^, *p <* 0.001

Interestingly, both sh-*lincSCRG1* + ov-SKP2 and sh-*lincSCRG1* + in-miR26a could effectively promote cell viability in the MTT assay (Fig. 6a), accelerate cell proliferation in the colony formation assay (Fig. 6b), decrease the frequency of cells in G1 phase and increase the frequency of cells in G2/S phase in the flow cytometry analysis (Fig. 6c), and elevate the migrating number of cells in the transwell assay (Fig. 6d) compared to sh-*lincSCRG1* in SNU-387 and Hep3B cells*.* In addition, downregulated pro-proliferation proteins (cyclin D1, CDK4/6) and EMT-related proteins (MMPs, N-cadherin and Vimentin) and upregulated E-cadherin by sh-lincSCRG1 could also be reversed by ov-SKP2 and in-miR26a (Fig. 6e, Fig. S2). The results proved that ov-SKP2 or in-miR26a could reverse these biological functions as a tumour suppressor by silencing *lincSCRG1* in HCC cells, displaying the contrasting results of sh-*lincSCRG1*. These data suggested that *lincSCRG1* promotes tumour cell growth, proliferation and migration by competitive inhibition of the target miR26a to elevate SKP2 levels, which involves the expression of proteins relevant to the cell cycle and EMT.

### Silencing *lincSCRG1* inhibits the growth and metastasis of HCC, which could be reversed by miR26a

**Fig. 7:**
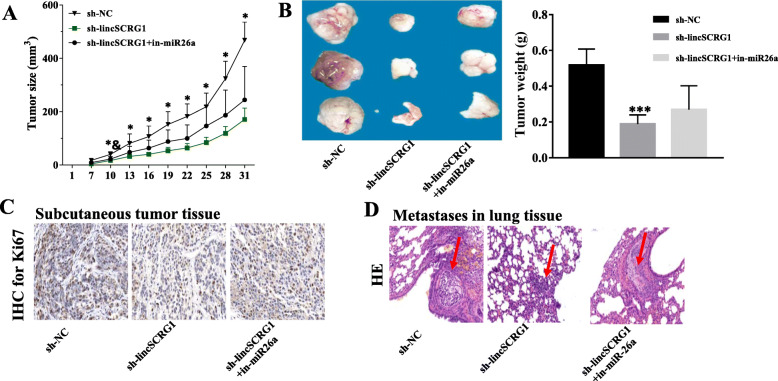
Silencing *LincSCRG1* inhibitsthe growth and metastasis of HCC, which could be reversed by miR26a. Nude mouse models were established with subcutaneous or intravenous injection of SNU-387 cells transfected with sh-NC, sh*-lincSCRG1* or sh*-lincSCRG1* + in-miR26a. Curves of subcutaneous tumour volume (**a**), tumour weight (**b**), and IHC staining for Ki-67 in tumour tissues (**c**) are displayed in tumour xenograft mice (each group, *n* = 3). **d** Lung metastasis tissue images (400 ×) after HE staining in the metastasis mouse model (each group, *n* = 3). In (**a**) ^*^indicatesthe sh-*lincSCRG1* vs. sh-NC group, *p <* 0.05; ^&^indicatesthe sh-*lincSCRG1* + in-miR26a vs. sh-*lincSCRG1* group, *p <* 0.05. In (**b**), *** indicatesthe sh-*lincSCRG1* vs. sh-NC group, *p <* 0.001

To explore the influence of *lincSCRG1* on the growth and metastasis of HCC in vivo, nude mouse models were established with subcutaneousor intravenous administration of SNU-387 cells transfected with sh-NC, sh*-lincSCRG1* or sh*-lincSCRG1* + in-miR26a. The tumour volume and weight of the nude mice were measured. Sh-*lincSCRG1* notably inhibited tumour growth, resulting in a significantly decreased tumour volume and weightafter the 10th day in the tumour xenograft mice compared to the sh-NC mice; however, in-miR26a substantially reversed the tumour suppressor effect of sh-*lincSCRG1* (Fig. 7a-b). In addition, IHC of Ki67 was employed to quantitively assess the proliferation index of the tissues from the xenograft tumours. Significantly fewer Ki67-positive cells were observed in the sh-*lincSCRG1* group of tumour xenograft mice than the sh-NC group, while dramatically more Ki67-positive cells were observed in the sh-*lincSCRG1* + in-miR26a mice than the sh-*lincSCRG1* mice (Fig. 7c). Furthermore, the size and number of lung metastatic nodules were visibly suppressed in the sh-*lincSCRG1* group of metastatic mice compared to the sh-NC group, while in-miR26a also reversed the impeded lung metastasis induced by sh-*lincSCRG1* (Fig. 7d). Collectively, these data suggested that silencing *lincSCRG1* could relieve tumour proliferation and metastasis in HCC, which was partially mediated by miR26a.

## Discussion

According to statistics, liver cancer is one of the most common malignancies worldwide, with the eighth highest incidence rate and fourth highest mortality rate [13]. HCC is the most common histologic subtype amongmolecular subtypes (90–95%) [14]. It is also regarded as the most common primary malignancy of the liver and a major cause of mortality [1]. However, its molecular mechanism remains a mystery; thus, identification of new molecular targets is needed to improve the therapeutic outcomes of HCC.

In recent years, a number of studies have reported that aberrant expression of lncRNAs is related to hepatocarcinogenesis [15–17]. LncRNAs, a heterogeneous group of RNA transcripts with a length of more than 200 nucleotides, are generally regarded as lacking protein-coding roles [3]. The classic types of lncRNAs are scaffold lncRNAs, decoy lncRNAs, signal lncRNAs and guide lncRNAs [18]. These diverse roles determine the complex functions, including coordination of the cell state, differentiation, development and involvement of diseases [19]. Our previous study revealed that*lincSCRG1*, a novel lncRNA, was upregulated along with the progression of liver fibrosis in humans by repressing the TTP-elicited inactivating effect on HSCs [6]. In this study, we found that *lincSCRG1* was elevated 2.31-fold in human HCC tissues compared to the adjacent liver tissues and approximately 1.84 ~ 4.46-fold in HCC cell lines compared to normal liver cell lines. Therefore, we hypothesized that *lincSCRG1* might participate in the pathogenesis of HCC. Thus, we established ov*-lincSCRG1* Hep3B cells and sh-*lincSCRG1* SNU-387 cells for MTT, colony formation, flow cytometry and transwell assays to evaluate the functions of *lincSCRG1* in HCC. Ov-*lincSCRG1* significantly promoted cell proliferation and migration, while sh-*lincSCRG1* presented the opposite effect. Decreases in cell cycle-related protein (cyclin D1, CDK4/6), invasion-related protein (MMP2/3/9) and adhesion-related protein (N-cadherin, Vimentin) expression levels and increases in E-cadherin expression levels were observed in the ov-*lincSCRG1* Hep3B cells, while the opposite results were observed in the sh-*lincSCRG1* SNU-387 cells. Furthermore, we established an HCC model of nude mice with *lincSCRG1* downregulation to assess the effect of *lincSCRG1* in vivo. We found that sh-*lincSCRG1* significantly decreased the tumour size and weight of subcutaneous neoplasms and inhibited lung metastasis of HCC. These findings indicate that *lincSCRG1* plays an oncogenic role in HCC by promoting cell proliferation, migration and EMT in vitro and in vivo.

LncRNAs were once considered “DNA junk” or “transcript noise”, and recent studies have revealed that they could be involved in transcriptional and post-transcriptional processes and epidemic networks to perform multiplex biological functions [20]. Currently, common regulatory mechanisms of lncRNAs on miRNAs, such as functioning as miRNA precursors [21], attenuating transcription factor gene expression and transactivation [22], facilitating primary-miRNA processing [23], and acting as “miRNA sponges” or “ceRNAs” to compete with miRNA response elements to block target miRNAs, have been confirmed [8, 9]. Among them, the latter mechanism has become a research hotspot. As *lincSCRG1*is an unknown noncoding RNA, we used the TargetScan algorithm to select a target ce-miRNA. We identified 7 miRNAs (miR1297, miR4465, miR26a/b, miR4779, miR4778, miR345 and miR203) that were expressed at low levels in tumour tissues or cells. Among them, miR1297/26a/26b/345/203 have been reported to be involved in HCC development. Only miR26a was significantly enhanced by sh-*lincSCRG1*but downregulated by ov-*lincSCRG1*. Both luciferase reporter and RIP assays further demonstrated that *lincSCRG1* had binding sites for miR26a and negatively regulated miR26a as a ceRNA. Therefore, we chose miR26a as the key target miRNA for further research. A multitude of studies have indicated that miR26a serves as a suppressor in the pathogenesis of HCC [10, 24, 25]. In our study, we also showed that inhibition of miR26a (in-miR26a) had similar effects as ov-*lincSCRG1*, while overexpression of miR26a (mi-miR26a) had similar effects as sh-*lincSCRG1*. In-miR26a also promoted cell proliferation and migration and decreased the protein levels of cyclin D1, CDK4/6, MMP2/3/9, N-cadherin, and Vimentin but increased the protein levels of E-cadherin in Hep3B cells, while mi-miR26a had the opposite effect in SNU387 cells. Furthermore, we verified that in-miR26a could counteract the protective effect of sh-*lincSCRG1*, including inhibition of tumour growth (tumour size or weight) in a xenograft tumour model and inhibition of lung metastasis in a mouse model. Thus, our results suggested that *lincSCRG1* functions as an oncogene in HCC by competing with miR26a to inhibit the tumour suppressor effect.

Recent studies have demonstrated that miR26a could exert anticancer functions in the progression of HCC through various mechanisms, including the miR26a-mRNA [24], lncRNA-miR26a-mRNA [10], and cicRNA-miR26a-mRNA axis [26] and regulation of fatty acid and cholesterol homeostasis [25]. By bioinformatics analysis, we identified an innovative target gene, SKP2, with eight binding sites in the 3’UTR for miR26a. Because the relationship between SKP2 and miR26a is still unknown, our study also confirmed that SKP2 is a downstream target of miR26a. Mi-miR26a distinctly decreased the SKP2 mRNA and protein levels in HCC cell lines. Only cotransfection with SKP2-wt and mi-miR26a decreased the luciferase activity, which was not observed with cotransfection with SKP2-mut. Then, we validated the regulatory function of *lincSCRG1* via the miR26a/SKP2 axis. Sh-*lincSCRG1* strongly downregulated SKP2 at both the mRNA and protein levels, while cotransfection with ov-SKP2 or in-miR26a abolished this effect. Therefore, we demonstrated the regulatory mechanism of the *lincSCRG1*/miR26a/SKP2 axis in HCC cells. SKP2 is a cell cycle-dependent protein, and it shows low expression in the early G1 phase but is increased from G1 to S phase [27]. A recent study revealed that SKP2 not only participated in the proliferation of HCC cells through the potassium channel KCa3.1 but was also upregulated in poorly differentiated HCC tissue [12]. Consistent with our results, upregulation of SKP2 was also found in HCC cells, and overexpression of SKP2 (ov-SKP2) could evidently rescue the antioncogenic function of sh-*lincSCRG1*, including promoting proliferation and migration of HCC cells. Moreover, the same effects were reflected in the elevated pro-proliferation proteins (cyclin D1, CDK4/6) and EMT-related proteins (MMPs, N-cadherin and Vimentin) and reduced E-cadherin with ov-SKP2. Therefore, we speculated that *lincSCRG1*/miR26a could finally promote the function of SKP2 in the cell cycle and stimulate EMT.

Interestingly, our study suggested that *lincSCRG1* is a common molecular regulator for the pathological processes of fibrosis [6] and cancer genesis. Tumor microenvironment, a dynamic system orchestrated by intercellular communications, is responsible for tumor progression and metastasis [28]. Many studies from the build co-culture model have shown that HCC cells co-cultured with HSCs increased tumor stemness [29], metastasis [30] and promoted HCC progression by formed the inflammatory liver cancer microenvironment. A switch from quiescent fibroblasts to cancer-associated fibroblasts (CAFs) triggers a large variety of pro-tumorigenic signals that support tumor progression and shape the surrounding pathological stroma, with the remodelling of tissue architecture and repression of the local immune response [31]. So, we speculated that *lincSCRG1* probably acts as a pro-fibrotic and pro-tumorigenic regulator to trigger HSCs and CAFs in the pathological progression from liver fibrosis to cirrhosis, and finally carcinogenesis. The multiple functions and mechanisms are subject of further active research.

## Conclusions

In summary, we showed that the *lincSCRG1*/miR26a/SKP2 axis plays an important role in HCC. Briefly, *lincSCRG1* could act as a ceRNA for miR26a to restrict its biological role to increase the expression of SKP2, thereby inducing the proliferation and migration of HCC in vitro and in vivo. To date, no drugs targeting lncRNAs have been applied. Our study indicated *lincSCRG1* depletion as a potential therapeutic approach in HCC. Further studies need to be performed to reveal the connection between *lincSCRG1* and other tumours to explore a new broad-spectrum antitumour target.

## Supplementary Information

**Additional file 1: Figure S1.** SKP2 protein expression in HCC cells. (A) The expressions of SKP2 proteins were reduced in mi-miR26a compared to mi-NC cells in both SNU-387 and Hep3B cell lines. (B) Co-transfected of ov-SKP2 or in-miR26a with sh-lincSCRG1could rescue the depletion of SKP2 protein inducing by sh-lincSCRG1 in 2 cell lines. **Figure.S2.** Different proteins expression in HCC cells. Sh-lincSCRG1 could down-regulate pro-proliferation related proteins (cyclin D1, CDK4/6) and ETM related proteins (MMPs, N-cadherin and Vimentin), and up-regulate E-cadherin, which could be reversed by co-transfection with ov-SKP2 and in-miR26a in SNU-387 and Hep3B cell lines.

**Additional file 2: Suppl Table 1.** Information of Plasmids used in study. **Suppl Table 2.** The sequence of related genes for qPCR. **Suppl Table 3.** The details of antibodies. **Suppl Table 4.** The putative microRNAs that may be regulated by linc-SCRG1.

## Data Availability

All data generated or analyzed during this study are included in this published article and its supplementary information files.

## Abbreviations

LncRNAs: Long noncoding RNAs
HCC: Hepatocellular carcinoma
SKP2: S phase kinase related protein 2
ceRNA: Competing endogenous RNA
*lincSCRG1*: Long noncoding RNA-SCRG1
miR-26a: microRNA-26a
ov-*lincSCRG1*: Overexpressing*lincSCRG1*
sh-*lincSCRG1*: Silencing *lincSCRG1*
ov-vector: Empty vector
sh-NC: shRNA negative control
mi-miR26a: miR-26a minic
in-miR26a: miR-26a inhibitor
in-NC: miRNA inhibitor negative control
mi-miRNA: miRNA negative control
ov-SKP2: Overexpressing SKP2
shRNA: Short hairpin RNA
EMT: Epithelial-to-mesenchymal transition
IHC: Immunohistochemistry
HE: Hematoxylin-eosin
qPCR: Quantitative real-time PCR
CDK4/6: Cyclin-dependent kinases 4/6
MMP2/3/9: Matrix metalloproteinases 2/3/9
MTT: The 3-(4,5-dimethylthiazol-2-yl)-2,5-diphenyltetrazolium bromide
OD: optical density
DMEM: Dulbecco’s modified Eagle’s medium
RIP: RNA Immunoprecipitation
HSCs: Hepatic stellate cells
*lincSCRG1*-wt: Wild type of *lincSCRG1*
*lincSCRG1*-mut: Mutant type of *lincSCRG1*
WNK1: With-no-lysine kinase 1
MAT2A: Methionine adenosyltransferase 2A
EIF5A2: Eukaryotic translation initiation factor 5 A2)
3’UTR: 3′-untranslatedregion
SKP2-wt: Wild type of SKP2
SKP2-mut: Mutant type of SKP2
CAFs: Cancer-associated fibroblasts
