# Correction to: *Linc-SCRG1* accelerates progression of hepatocellular carcinoma as a ceRNA of miR26a to derepress SKP2

**DOI:** 10.1186/s13046-021-01975-x

**Published:** 2021-05-25

**Authors:** Jun-Jie Hu, Cui Zhou, Xin Luo, Sheng-Zheng Luo, Zheng-Hong Li, Zi-Xin Xu, Ming-Yi Xu

**Affiliations:** Department of Gastroenterology, Shanghai General Hospital, Shanghai Jiao Tong University School of Medicine, No 100, Haining Rd, Shanghai, 200080 China

**Correction to: J Exp Clin Cancer Res 40, 26 (2021)**

**https://doi.org/10.1186/s13046-020-01825-2**

Following publication of the original article [1], the authors identified minor errors in image-typesetting in Fig. 2, Fig. 4 and Fig. 6; specifically:

- Figure 2E: the western blot figure of GAPDH of SNU-387 (row 4) was incorrectly used; the correct image has now been used
- Figure 2E: the label of groups of western blot was incorrect; the correct label has now been used
- Figure 4B: during the production process, image distortion was introduced in the colony study of Hep3B cells; this has been corrected using the originally provided image files
- Figure 4E: the western blot figure of E-cadherin of SNU-387 was incorrectly used; the correct image has now been used
- Figure 4E: the label of groups of western blot was incorrect; the correct label has now been used
- Figure 6B: during the production process, image distortion was introduced in the colony study of Hep3B cells; this has been corrected using the originally provided image files
- Figure 6D: the transwell figures of sh-*lincSCRG1* + ov-SKP2 (row 3) and sh-*lincSCRG1* + in-miR26a (row 4) groups of SNU-387 and Hep3B were incorrectly used; the correct images have now been used

**Fig. 2:**
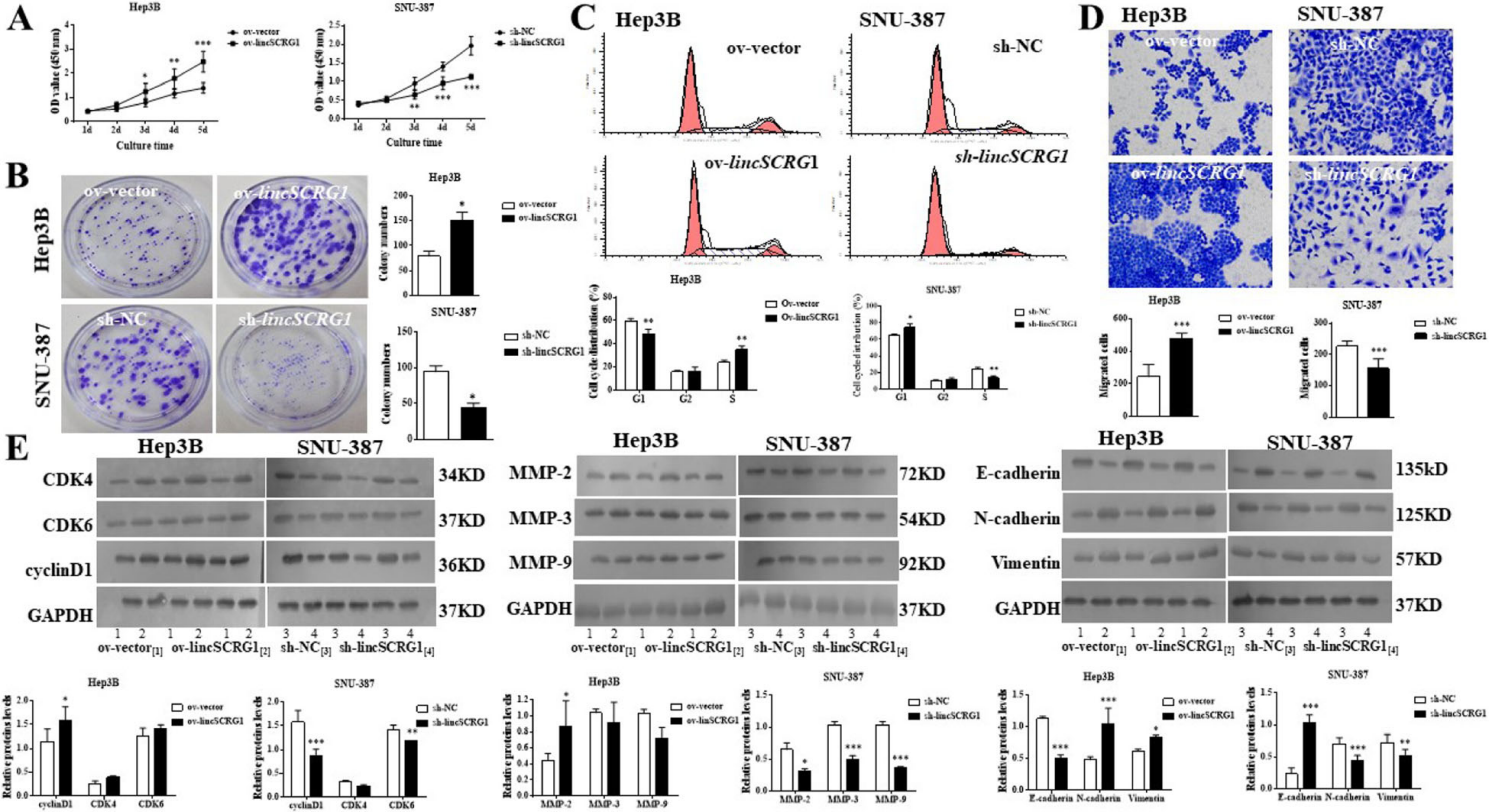
Overexpression of lincSCRG1 dramatically promoted HCC cell proliferation and migration in vitro. Sh-lincSCRG1, sh-NC (in SNU-387 cells), ov-lincSCRG1 and ov-vector (in Hep3B cells) cell lines were established. **a** Cell viability was examined by MTT assays. **b** Oncogenic survival was assessed by colony formation assays. **c** Cell cycle proliferation was evaluated by flow cytometry. **d** Migration was determined by transwell assays. **e** Cell cycle-related proteins (CKD4/6 and cyclinD1) and EMT-related proteins (MMP-2/3/9, E-cadherin, N-cadherin and Vimentin) were examined by western blot analysis. In (**a**-**e**), */**/***indicates vs. The ov-vector/sh-NC group (*, *p* < 0.05, **, *p* < 0.01, ***, *p* < 0.001)

**Fig. 4:**
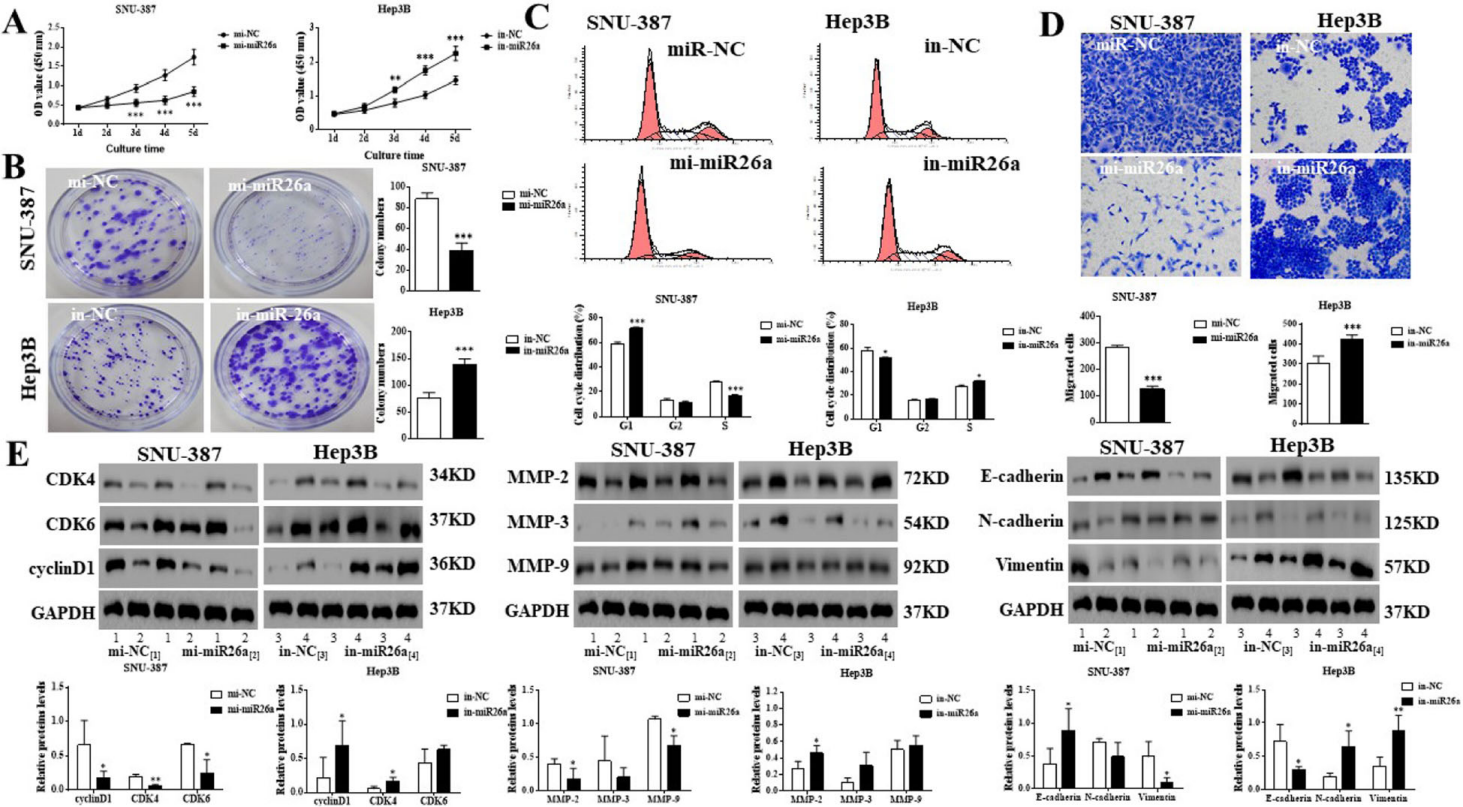
MiR26a is negatively correlated with the proliferation and migration of HCC in vitro. Mi-miR26a, mi-NC (in SNU-387 cells), in-miR26a and in-NC (in Hep3B cells) cell lines were established. **a** Cell viability was examined by MTT assays. **b** Oncogenic survival was assessed by colony formation assays. **c** Cell cycle proliferation was evaluated by flow cytometry. **d** Migration was determined by transwell assays. **e** Cell cycle-related proteins (CKD4/6 and cyclinD1) and EMT-related proteins (MMP-2/3/9, E-cadherin, N-cadherin and Vimentin) were examined by western blot analysis. In (**a-e**), */**/***indicates vs. The mi-NC/in-NC group (*, *p* < 0.05, **, *p* < 0.01, ***, *p* < 0.001)

**Fig. 6:**
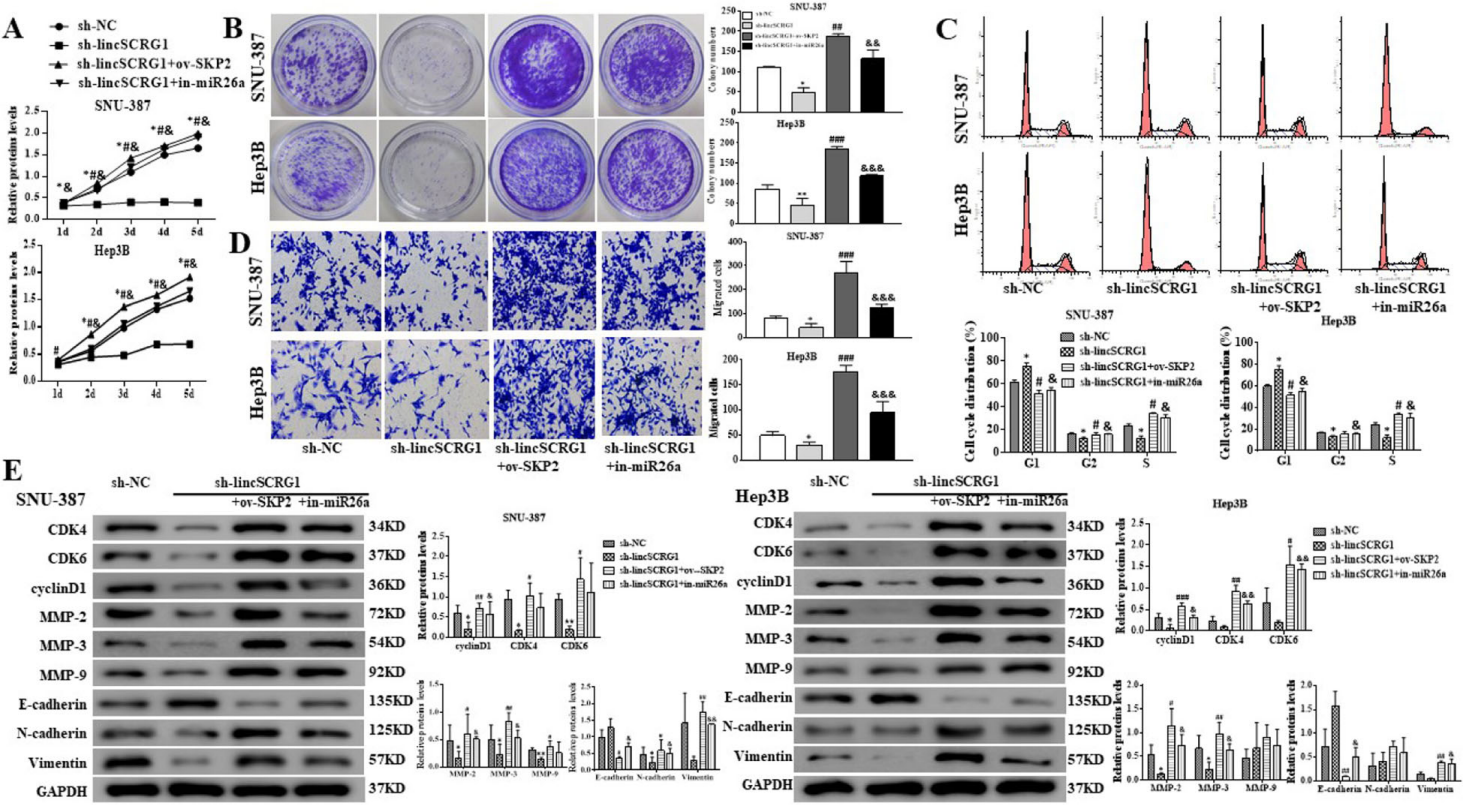
*LincSCRG1* promotes cell proliferation and migration of HCC via regulating the miR26a/SKP2 axis in vitro. Sh-NC, sh-*lincSCRG1*, sh-*lincSCRG1* + ov-SKP2 and sh-*lincSCRG1* + in-miR26a groups were established in SNU-387 and Hep-3B cell lines. **a** Cell viability was examined by MTT assays. **b** Oncogenic survival was assessed by colony formation assays. **c** Cell cycle proliferation was evaluated by flow cytometry. **d** Migration was determined by transwell assays. **e** Cell cycle-related proteins (CKD4/6 and cyclinD1) and EMT-related proteins (MMP-2/3/9, E-cadherin, N-cadherin and Vimentin) were examined by western blot analysis. In (**a - d**) ^*/#/&^indicatesthe sh-*lincSCRG1* vs. sh-NC group, the sh-*lincSCRG1* + ov-SKP2 vs. sh-*lincSCRG1* -group, and the sh-*lincSCRG1* + in-miR26a vs. sh-*lincSCRG1* group, respectively (*n* = 6). ^* /#/&^, *p* < 0.05, ^**/##/&&^, *p* < 0.01, ^***/###/&&&^, *p* < 0.001

The corrected figures are given below. The corrections do not have any effect on the results or conclusions of the paper. The original article has been corrected.
