# Supplementary material for: Linc-SCRG1 accelerates progression of hepatocellular carcinoma as a ceRNA of miR26a to derepress SKP2
Source: J Exp Clin Cancer Res. 2021 Jan 9;40:26. doi: 10.1186/s13046-020-01825-2 (PMC7797122; doi:10.1186/s13046-020-01825-2)
Supplement: Supplementary file 1 — Additional file 1: Figure S1. SKP2 protein expression in HCC cells. (A) The expressions of SKP2 proteins were reduced in mi-miR26a compared to mi-NC cells in both SNU-387 and Hep3B cell lines. (B) Co-transfected of ov-SKP2 or in-miR26a with sh-lincSCRG1could rescue the depletion of SKP2 protein inducing by sh-lincSCRG1 in 2 cell lines. Figure.S2. Different proteins expression in HCC cells. Sh-lincSCRG1 could down-regulate pro-proliferation related proteins (cyclin D1, CDK4/6) and ETM related proteins (MMPs, N-cadherin and Vimentin), and up-regulate E-cadherin, which could be reversed by co-transfection with ov-SKP2 and in-miR26a in SNU-387 and Hep3B cell lines. [file 13046_2020_1825_MOESM1_ESM.pptx]

## Slide 1
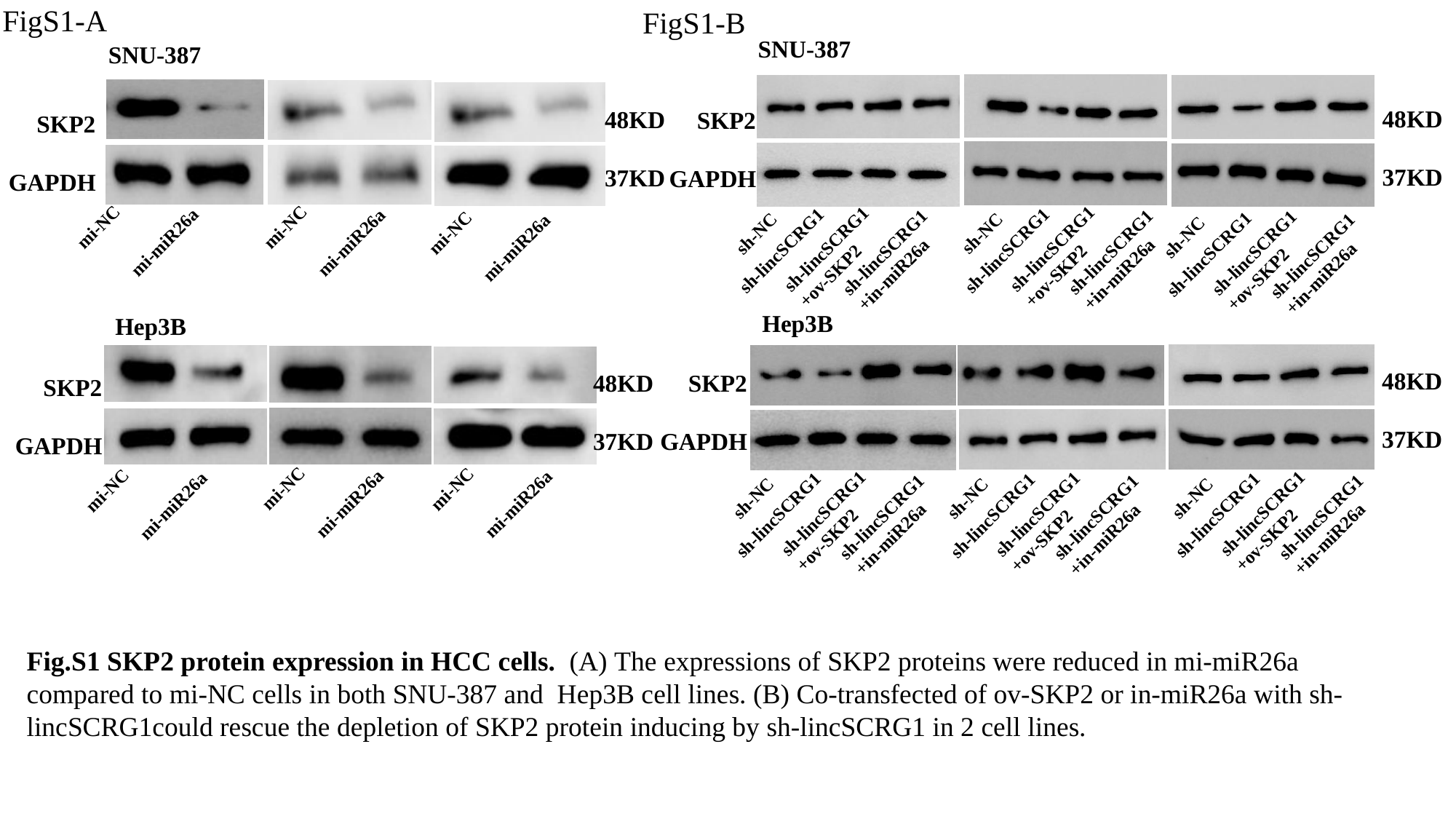

FigS1-A
FigS1-B
SNU-387
SNU-387
48KD
37KD
48KD
37KD
SKP2
GAPDH
SKP2
GAPDH
mi-miR26a
 mi-NC
mi-miR26a
 mi-NC
mi-miR26a
 mi-NC
sh-lincSCRG1
+in-miR26a
sh-lincSCRG1
+ov-SKP2
sh-NC
sh-lincSCRG1
sh-lincSCRG1
+in-miR26a
sh-lincSCRG1
+ov-SKP2
sh-NC
sh-lincSCRG1
sh-lincSCRG1
+in-miR26a
sh-lincSCRG1
+ov-SKP2
sh-NC
sh-lincSCRG1
Hep3B
Hep3B
48KD
37KD
48KD
37KD
SKP2
GAPDH
SKP2
GAPDH
mi-miR26a
 mi-NC
mi-miR26a
 mi-NC
mi-miR26a
 mi-NC
sh-lincSCRG1
+in-miR26a
sh-lincSCRG1
+ov-SKP2
sh-NC
sh-lincSCRG1
sh-lincSCRG1
+in-miR26a
sh-lincSCRG1
+ov-SKP2
sh-NC
sh-lincSCRG1
sh-lincSCRG1
+in-miR26a
sh-lincSCRG1
+ov-SKP2
sh-NC
sh-lincSCRG1
Fig.S1 SKP2 protein expression in HCC cells. (A) The expressions of SKP2 proteins were reduced in mi-miR26a compared to mi-NC cells in both SNU-387 and Hep3B cell lines. (B) Co-transfected of ov-SKP2 or in-miR26a with sh-lincSCRG1could rescue the depletion of SKP2 protein inducing by sh-lincSCRG1 in 2 cell lines.

## Slide 2
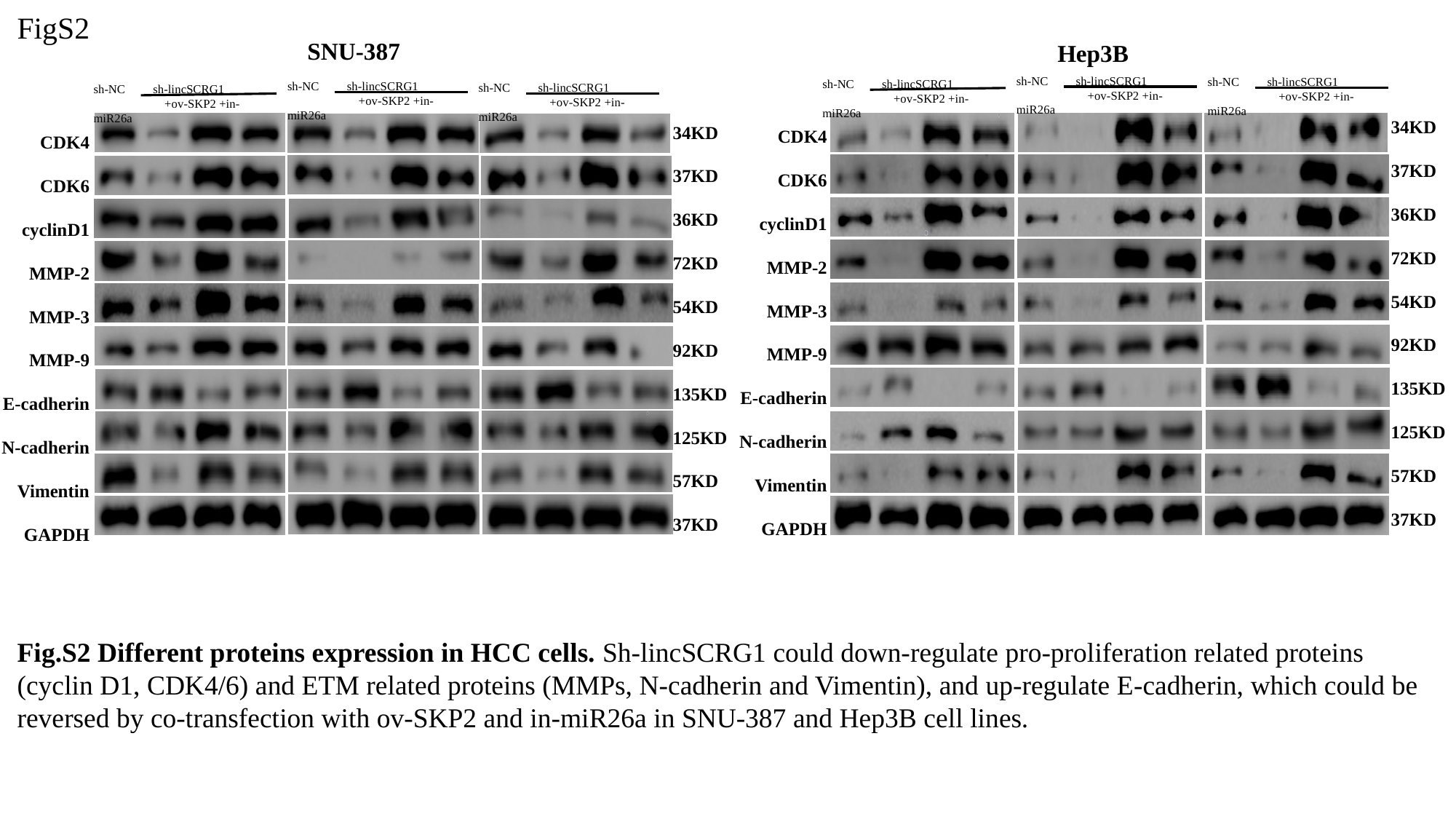

FigS2
SNU-387
Hep3B
sh-NC sh-lincSCRG1
 +ov-SKP2 +in-miR26a
sh-NC sh-lincSCRG1
 +ov-SKP2 +in-miR26a
sh-NC sh-lincSCRG1
 +ov-SKP2 +in-miR26a
sh-NC sh-lincSCRG1
 +ov-SKP2 +in-miR26a
sh-NC sh-lincSCRG1
 +ov-SKP2 +in-miR26a
sh-NC sh-lincSCRG1
 +ov-SKP2 +in-miR26a
CDK4
CDK6
cyclinD1
MMP-2
MMP-3
MMP-9
E-cadherin
N-cadherin
Vimentin
GAPDH
CDK4
CDK6
cyclinD1
MMP-2
MMP-3
MMP-9
E-cadherin
N-cadherin
Vimentin
GAPDH
34KD
37KD
36KD
72KD
54KD
92KD
135KD
125KD
57KD
37KD
34KD
37KD
36KD
72KD
54KD
92KD
135KD
125KD
57KD
37KD
Fig.S2 Different proteins expression in HCC cells. Sh-lincSCRG1 could down-regulate pro-proliferation related proteins (cyclin D1, CDK4/6) and ETM related proteins (MMPs, N-cadherin and Vimentin), and up-regulate E-cadherin, which could be reversed by co-transfection with ov-SKP2 and in-miR26a in SNU-387 and Hep3B cell lines.
